# Modulation of Telomerase Activity in Cancer Cells by Dietary Compounds: A Review

**DOI:** 10.3390/ijms19020478

**Published:** 2018-02-06

**Authors:** Takahiro Eitsuka, Kiyotaka Nakagawa, Shunji Kato, Junya Ito, Yurika Otoki, Soo Takasu, Naoki Shimizu, Takumi Takahashi, Teruo Miyazawa

**Affiliations:** 1 Food & Biodynamic Chemistry Laboratory, Graduate School of Agricultural Science, Tohoku University, Sendai 980-0845, Japan; nkgw@m.tohoku.ac.jp (K.N.); tyunkato@gmail.com (S.K.); junyai@tohoku.ac.jp (J.I.); fieldlily.716@gmail.com (Y.O.); soo.takasu@dc.tohoku.ac.jp (S.T.); nshimizu@dc.tohoku.ac.jp (N.S.); t-u.tak@dc.tohoku.ac.jp (T.T.); 2 Food and Biotechnology Innovation Project, New Industry Creation Hatchery Center (NICHe), Tohoku University, Sendai 980-8579, Japan; miyazawa@m.tohoku.ac.jp; 3 Food and Health Science Research Unit, Graduate School of Agricultural Science, Tohoku University, Sendai 980-0845, Japan

**Keywords:** telomerase, hTERT, cancer, dietary compound

## Abstract

Telomerase is expressed in ~90% of human cancer cell lines and tumor specimens, whereas its enzymatic activity is not detectable in most human somatic cells, suggesting that telomerase represents a highly attractive target for selective cancer treatment. Accordingly, various classes of telomerase inhibitors have been screened and developed in recent years. We and other researchers have successfully found that some dietary compounds can modulate telomerase activity in cancer cells. Telomerase inhibitors derived from food are subdivided into two groups: one group directly blocks the enzymatic activity of telomerase (e.g., catechin and sulfoquinovosyldiacylglycerol), and the other downregulates the expression of human telomerase reverse transcriptase (hTERT), the catalytic subunit of human telomerase, via signal transduction pathways (e.g., retinoic acid and tocotrienol). In contrast, a few dietary components, including genistein and glycated lipid, induce cellular telomerase activity in several types of cancer cells, suggesting that they may be involved in tumor progression. This review summarizes the current knowledge about the effects of dietary factors on telomerase regulation in cancer cells and discusses their molecular mechanisms of action.

## 1. Introduction

Telomeres cap the ends of linear chromosomes and maintain chromosomal stability by preventing end-to-end fusion and degradation [1]. In mammals, telomeres are composed of 5′-TTAGGG-3′ tandem base pair repeats followed by a 3′ G-rich single-stranded overhang. Because conventional DNA polymerases cannot completely replicate the 3′ end of lagging strands owing to the *end-replication problem*, telomeres shorten by ~50–200 bases with each round of cell division [2]. Eventually, telomeres reach a critical length and cells enter replicative senescence (the so-called Hayflick limit). Human telomerase, a ribonucleoprotein enzyme, elongates the 3′ end of telomeres by adding the tandem arrays of TTAGGG repeats [3]. Telomerase is expressed in ~90% of cancer cells and tumor tissues [4], indicating that the addition of telomeric DNA by telomerase contributes to the infinite proliferation of cancer cells. The tumor cells without telomerase activity utilize a unique mechanism of telomere maintenance termed *alternative lengthening of telomeres* to keep dividing without limits [5]. In most somatic cells, this enzymatic activity is not detectable [4]. Thus, normal cells have a limited ability to proliferate. On the other hand, germ cells [6] and self-renewing tissues (e.g., the ovary [4], intestinal epithelium [7], and hematopoietic stem cells [8]) possess telomerase activity.

Human telomerase is essentially composed of two subunits: human telomerase RNA component (hTR; also known as hTERC) and human telomerase reverse transcriptase (hTERT). hTR contains an RNA template complementary to the 3′ overhang of telomeres [9]. hTERT acts as the catalytic subunit that adds telomeric DNA to the 3′ overhang [10,11]. Besides, some telomerase-associated proteins such as dyskerin, NOP10, NHP2, and GAR1 have been reported to contribute to proper telomerase function in vivo [12]. Although hTR and telomerase-associated proteins are ubiquitously expressed in all human cells, hTERT is expressed only in telomerase-positive cells and tissues. The expression level of *hTERT* mRNA highly correlates with cellular telomerase activity [13], suggesting that hTERT is a critical determinant of telomerase activity. Thus, it is important to understand the mechanism underlying hTERT regulation in order to take advantage of telomerase for cancer diagnosis and treatment.

**Figure 1:**
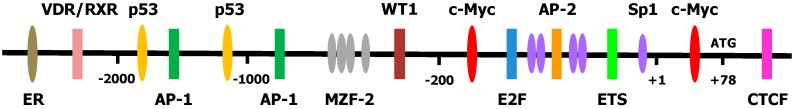
Promoter region of *hTERT* and transcription factors essential for regulating its activity. +1 is the transcription start site; +78 (ATG) is the first codon of the hTERT protein.

The 5′ promoter region of *hTERT* has been cloned [14,15,16], and several transcription factors, including c-Myc, Sp1, activating enhancer-binding protein-2 (AP-2), hypoxia-inducible factor 1 (HIF-1), ETS, estrogen receptor (ER), E2F, activator protein 1 (AP-1), vitamin D receptor (VDR) in complex with retinoid X receptor (RXR), p53, Wilms’ tumor 1 (WT1), myeloid zinc finger protein 2 (MZF-2), and CCCTC-binding factor (CTCF), have been found to modulate the transcriptional activity of the *hTERT* promoter (Figure 1) [17]. Among these transcription factors, c-Myc plays a pivotal role in *hTERT* mRNA expression. c-Myc can recognize and bind to the E-box consensus sequence (5′-CACGTG-3′) in the target gene promoter. The *hTERT* promoter contains two E-box sites located at positions −165 and +44 relative to the transcription start site (+1 position). The expression of *c-myc* mRNA is highly related to the expression of *hTERT* mRNA [18]. Treatment with antisense *c-myc* oligonucleotides downregulates *c-myc* and hTERT expression in human leukemic cells, thus leading to the suppression of cellular telomerase activity [19]. The above findings clearly indicate that c-Myc acts as a crucial signaling factor in the regulation of *hTERT* transcription.

**Figure 2:**
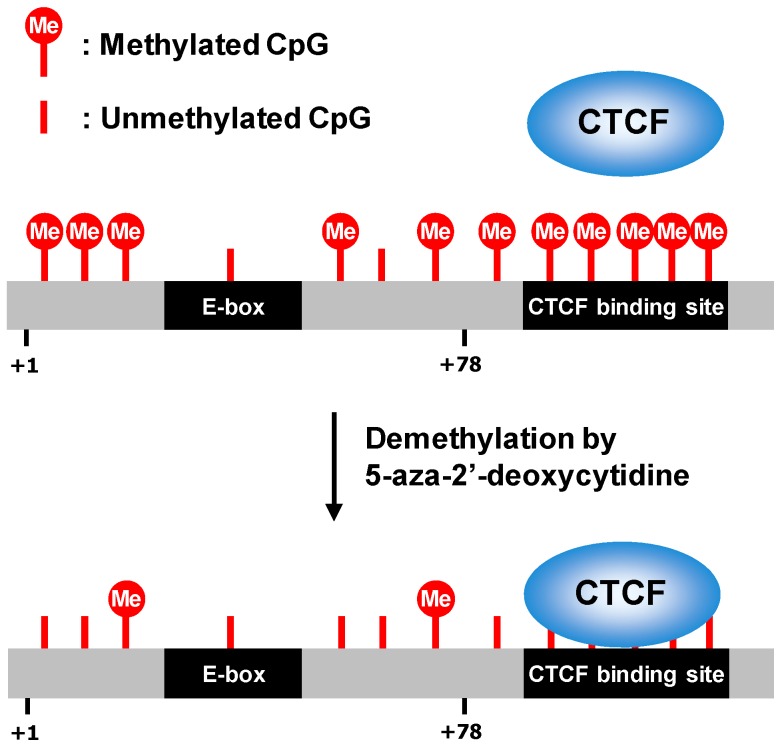
Regulation of *hTERT* transcription by 5-aza-2′-deoxycytidine. In cancer cells, CTCF recognition sequence in *hTERT* gene is hypermethylated, thus blocking CTCF binding and inducing *hTERT* expression. Treatment with 5-aza-2′-deoxycytidine leads to demethylation of *hTERT* promoter, thereby causing the binding of CTCF to the target site and suppressing *hTERT* transcription.

Epigenetic modifications (i.e., DNA methylation, histone modifications, and noncoding RNA) alter the recruitment of transcription factors and chromatin structure, thereby affecting gene expression [20]. DNA methylation is catalyzed by DNA methyltransferases (DNMTs) through the addition of a methyl group at the C-5 position of cytosine. These enzymes include DNMT1, DNMT3A, and DNMT3B; DNMT1 participates in the methylation of hemimethylated DNA to maintain the methylation status of daughter strands after the replication process, whereas DNMT3A and DNMT3B are responsible for de novo DNA methylation [21]. The cytosine methylation generally occurs in cytosine–guanine (CG) clusters (referred to as CpG islands). The methyl group in CpG islands is thought to directly block DNA recognition and binding by several transcription factors, meaning that CpG methylation is involved in gene silencing. The *hTERT* promoter contains many CpG sites, but most CpG islands in this promoter are surprisingly hypermethylated (especially from position −650 to −150 relative to the transcription start site) in telomerase-positive cancer cell lines [22]. Such hypermethylation of the *hTERT* promoter abrogates binding of transcriptional repressors, such as CTCF and E2F. In fact, treatment of cancer cells with 5-aza-2′-deoxycytidine, a DNA methylation inhibitor, allows CTCF to bind to the *hTERT* promoter and to repress *hTERT* expression (Figure 2) [23]. Thus, CpG hypermethylation of the *hTERT* promoter in tumor cells is intended in part to block binding of the transcriptional repressor, thereby increasing the transcription of the *hTERT* gene.

Histone modifications such as acetylation, methylation, phosphorylation, and ubiquitination participate in changes of chromatin structure, leading to altered gene transcription [24]. For instance, histone acetylation is mediated by histone acetyltransferases (HATs), which add an acetyl group to a lysine residue within a histone tail. The presence of acetylated lysine in the histone tail attenuates the interaction between histones and DNA, eliciting a relaxed chromatin structure thereby permitting access of transcription factors to the DNA and, therefore, promoting a transcriptionally active gene status. In contrast, histone deacetylases (HDACs) can remove the acetyl group and cause chromatin condensation and gene silencing. Some researchers have shown that trichostatin A (TSA), an HDAC inhibitor, induces *hTERT* mRNA expression and telomerase activity in a Mad- and Sp1-dependent manner in normal cells [25,26] but has no effect on cancer cells, such as human fibrosarcoma (HT-1080) and cervical cancer (HeLa and C33A) cell lines. In contrast, *hTERT* transcription and telomerase activity are suppressed by treatment with TSA in prostate cancer [27], leukemia [28], and brain cancer cells [29]. Choi et al. [30] revealed that TSA downregulates DNMT1 and causes demethylation of a CTCF-binding site in the *hTERT* promoter, resulting in repression of *hTERT* expression in HCT116 colorectal cancer cells. These results suggest that the effects of TSA on *hTERT* transcription depend on cell type. In addition to histone acetylation, *hTERT* expression is governed by histone methylation. SET and MYND domain-containing protein 3 (SMYD3) is a lysine 4 on histone 3 (H3K4)-specific dimethyl transferase and trimethyl transferase. SMYD3 binds to a specific DNA sequence (5′-CCCTCC-3′) in the promoter region of *hTERT*, facilitates dimethylation or trimethylation of H3K4, and activates *hTERT* transcription [31].

Noncoding RNAs perform key functions in epigenetic regulation. MicroRNAs (miRNAs), one of the largest families of noncoding RNAs, are ~23 nucleotides long [32]. They can post-transcriptionally downregulate or degrade their target mRNAs by binding to the recognition sites in the 3′ untranslated region (3′UTR) of their specific mRNA, thus acting as repressors of gene expression. Multiple types of miRNAs have been shown to target *hTERT* mRNA, thereby reducing proliferation of cells derived from neuroblastoma [33], anaplastic thyroid carcinoma [34], gastric cancer [35,36], cervical cancer [37], head and neck squamous cell carcinoma [38], ovarian cancer [39], and breast cancer (Table 1) [40]. These observations indicate that the epigenetic regulation of DNA methylation, histone modifications, and noncoding RNAs markedly contribute to *hTERT* gene expression.

Telomerase activity in cancer cells is also regulated by promoter mutations and rearrangements of *hTERT*. Point mutations in the *hTERT* promoter can generate new consensus motifs for transcription factors [41]. Structural rearrangements of *hTERT* bring the enhancers close to the *hTERT* promoter [42]. Therefore, mutations and rearrangements of *hTERT* participate in the upregulation of *hTERT* expression.

To date, we and other groups have found that several dietary compounds can regulate telomerase activity in cancer cells. Telomerase inhibitors derived from food are subdivided into two groups: one group interacts with telomerase and directly blocks its enzymatic activity, and the other downregulates the expression of *hTERT* via signal transduction pathways. On the other hand, a few dietary components upregulate cellular telomerase activity in several types of cancer cells, implying that they may be involved in tumor progression. This review summarizes the current knowledge about the effects of food factors on telomerase activity in cancer cells and discusses their molecular mechanisms of action.

## 2. Inhibition of Telomerase Activity by Dietary Components

### 2.1. Retinoic Acid

**Figure 3:**
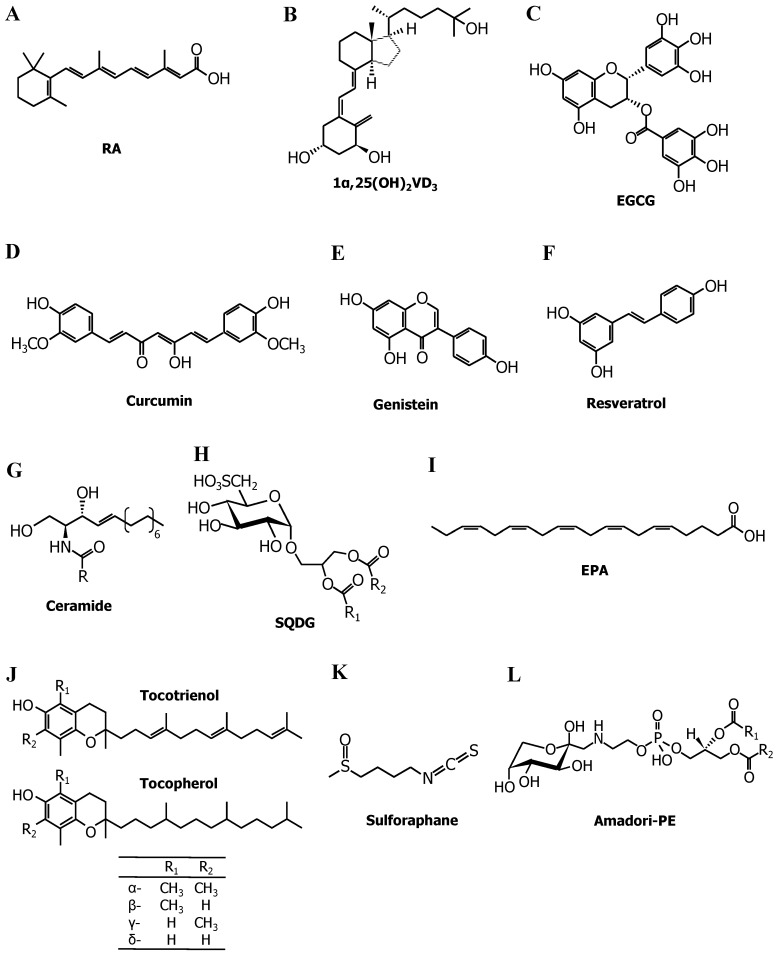
Chemical structures of telomerase modulators. (**A**) All-*trans* retinoic acid (RA). (**B**) 1α,25-Dihydroxyvitamin D_3_ (1α,25(OH)_2_VD_3_). (**C**) (−)-Epigallocatechin-3-gallate (EGCG). (**D**) Curcumin. (**E**) Genistein. (**F**) Resveratrol. (**G**) Ceramide. R, acyl chain. (**H**) Sulfoquinovosyldiacylglycerol (SQDG). R_1_ and R_2_ indicate acyl chains. (**I**) Eicosapentaenoic acid (EPA). (**J**) Tocotrienol (T3) and tocopherol (Toc). (**K**) Sulforaphane. (**L**) Phosphatidylethanolamine-linked Amadori product (Amadori-PE). R_1_ and R_2_ indicate acyl chains.

Vitamin A (also known as retinol) is a fat-soluble vitamin necessary for embryonic development, organ formation, immune function, and vision [43]. Retinol is converted to retinal by alcohol dehydrogenase in the body, and retinal can be altered to retinoic acid by retinaldehyde dehydrogenase. Retinoic acid, particularly all-*trans* retinoic acid (RA; Figure 3A), participates in the regulation of cell growth and differentiation [44]. One in vivo study showed that telomerase activity is reduced during stem cell maturation in embryonic development [45], suggesting that the suppression of telomerase may be linked to cell differentiation. Sharma et al. [46] investigated the effect of differentiation inducers, including dimethyl sulfoxide and RA (1 µM), on telomerase activity in HL-60 human leukemia cells, and demonstrated that both inducers decrease this enzymatic activity. These agents do not directly inhibit this activity at all. Furthermore, treatment of SW480 colon carcinoma cells with these inducers does not cause cell differentiation and has no effect on telomerase activity. Love et al. [47] showed that 2 µM RA induces differentiation of HL-60 cells and attenuation of *hTERT* expression via epigenetic regulation. Treatment with RA leads to hypoacetylation and hypermethylation of the *hTERT* promoter by altering DNMT expression. These results [46,47] indicate that telomerase inhibition takes place in response to the differentiation status. In contrast, Pendino et al. [48,49] reported that RA can downregulate telomerase independently of cell differentiation. In maturation-resistant NB4 acute promyelocytic leukemia cells, the reduction in telomerase activity by RA stems from the downregulation of *hTERT* mRNA expression through activation of retinoic acid receptor (RAR) and RXR [49]. However, 1 nM RA elicits telomerase activity and decreases p16^INK4A^ expression, therefore extending the lifespan of normal human oral keratinocytes, while 100 nM RA does not influence the cellular senescence [50]. It is noteworthy that the very low concentration of RA (1 nM) has the characteristic effect on the proliferative capacity of normal keratinocytes.

### 2.2. Vitamin D_3_

This vitamin is made in the skin from 7-dehydrocholesterol during exposure to ultraviolet sunlight. Vitamin D_3_ is metabolized by cytochrome P450 (CYP) enzymes such as 27A1 and CYP2R1 in the liver and is then converted to 1α,25-dihydroxyvitamin D_3_ (1α,25(OH)_2_VD_3_; Figure 3B), the physiologically active form of vitamin D_3_, by CYP27B1 in the kidneys [51]. 1α,25(OH)_2_VD_3_ is essential for bone remodeling, immunity, insulin secretion, and blood pressure regulation [52]. In addition to these beneficial effects, 1α,25(OH)_2_VD_3_ acts as a differentiation inducer as well as RA does. Treatment with 1α,25(OH)_2_VD_3_, therefore, drives the differentiation of HL-60 cells and suppresses telomerase activity [46]. Most physiological functions of 1α,25(OH)_2_VD_3_ are mediated by a nuclear transcription factor, VDR [53]. 1α,25(OH)_2_VD_3_ binds to VDR and promotes the interaction with its heterodimer partner, RXR, that is activated by 9-*cis*-retinoic acid. The VDR–RXR complex binds to a specific DNA sequence called a vitamin D response element (VDRE; there are several versions), initiating the transcription of target genes. Because the promoter region of *hTERT* contains a VDRE, Ikeda et al. [54] examined the influence of 1α,25(OH)_2_VD_3_ and 9-*cis*-retinoic acid on telomerase activity. Although treating PC3 human prostate cancer cells with a single treatment of 1α,25(OH)_2_VD_3_ or 9-*cis*-retinoic acid (10 nM each) did not inhibit telomerase, the combination of 1α,25(OH)_2_VD_3_ and 9-*cis*-retinoic acid reduced the enzymatic activity via direct interaction of the heterodimer of VDR and RXR with the VDRE. Significant suppression of tumor growth in nude mice inoculated with PC3 cells was observed after intraperitoneal injections of both 1α,25(OH)_2_VD_3_ (5 ng/mouse) and 9-*cis*-retinoic acid (5 ng/mouse) at 3-day intervals. Kasiappan et al. [55] provided direct evidence for the involvement of a miRNA, miR-498, in the downregulation of *hTERT* through VDR. 1α,25(OH)_2_VD_3_ (100 nM) causes the formation of the VDR–RXR heterodimer and its binding to a VDRE located in the 5′ regulatory region of the miR-498 gene in OVCAR3 human ovarian cancer cell line. miR-498 binds to the complementary sequence in the 3′UTR of *hTERT* mRNA, thereby leading to its breakdown. These findings imply that VDR plays a unique role in telomerase inhibition by 1α,25(OH)_2_VD_3_.

### 2.3. Polyphenols

These are a major group of phytochemicals present in fruits, vegetables, and beverages, with over 8000 phenolic structures currently identified [56].

The predominant polyphenols in green tea include (−)-epicatechin, (−)-epicatechin-3-gallate, (−)-epigallocatechin, and (−)-epigallocatechin-3-gallate (EGCG; Figure 3C), the last of which accounts for approximately 50% of the total phenolic content of green tea extract [57]. Naasani et al. [58] revealed that a physiological concentration of catechin (~1 μM), particularly EGCG, directly inhibits telomerase activity. An analysis of inhibition kinetics by means of a Dixon plot showed that EGCG is a competitive inhibitor relative to a telomerase substrate primer, indicating that EGCG competitively interacts with the substrate-binding site of telomerase. Prolonged treatment with 15 μM EGCG drives telomere shortening in U937 monoblastoid leukemia cells and HT29 adenocarcinoma cells and eventually induces senescence-associated β-galactosidase activity, a biomarker of cellular aging. Oral administration of EGCG (1.2 mg/mouse per day) significantly reduces tumor size in nude mice carrying an HCT-L1 (human colon carcinoma cells) xenograft [59]. In addition to direct inhibition of telomerase enzymatic activity, (−)-epigallocatechin and EGCG (20–40 μM) repress telomerase via downregulation of *hTERT* mRNA [60]. Berletch et al. [61] revealed that 100 μM EGCG causes demethylation of *hTERT* promoter and binding of E2F-1, an *hTERT* repressor, to the target promoter in MCF-7 breast cancer cells. Furthermore, Min et al. [62] showed that the decrease in *hTERT* mRNA expression by EGCG in HCT116 human colon cancer cells is driven by increased binding of CTCF to the *hTERT* core promoter region via downregulation of DNMT1 expression. Two mechanisms for DNMT inhibition by EGCG have been proposed: EGCG inhibits DNMT activity via direct binding to the catalytic site [63]; alternatively, the suppression of DNMT mRNA expression is implicated in the p21^WAF1^–p300–DNMT axis [62,64]. Therefore, the transcriptional repression of *hTERT* is primarily due to epigenetic regulation by EGCG.

Curcumin (Figure 3D) is a major component of turmeric and is commonly consumed as a spice in Asian countries. Curcumin affects several signaling molecules, thus exerting physiological effects such as antioxidant, anti-inflammatory, anticancer, and neuroprotective [65]. Ramachandran et al. [66] revealed that treatment with 50–100 μM curcumin decreases telomerase activity and *hTERT* mRNA expression in MCF-7 cells but does not influence *c-myc* mRNA. This result means that curcumin downregulates telomerase through a c-Myc–independent pathway. Heat shock protein 90 (Hsp90), a molecular chaperone, performs essential functions in the folding and maturation of its substrate proteins, and p23 is a co-chaperone that stabilizes Hsp90-substrate protein complexes. Hsp90 and p23 can bind to hTERT and promote its proper assembly with hTR [67]. Lee and Chung [68] discovered that curcumin disrupts the binding of p23 to hTERT, attenuating nuclear translocation of the hTERT protein and thereby inhibiting telomerase activity. Khaw et al. [69] demonstrated that curcumin prevents telomerase activity and *hTERT* expression and found that prolonged treatment (15 days) causes telomere shortening in brain tumor cells.

Genistein (Figure 3E), an isoflavone found abundantly in soy, has protective effects against cancer, obesity, osteoporosis, and inflammation [70]. Some reports have proven that phosphorylation of the hTERT subunit by the Akt kinase [71] and protein kinase C (PKC) [72] augments telomerase activity, indicating that telomerase can be controlled by post-translational modifications. Jagadeesh et al. [73] reported that 10–100 μM genistein reduces telomerase activity in DU-145 and PC-3 prostate cancer cells not only by decreasing *hTERT* expression through downregulation of *c-myc* but also by dephosphorylation of hTERT through Akt inhibition. Li et al. [74] revealed that treatment of MCF-7 cells with 50–100 μM genistein inhibits DNMT expression and causes hypomethylation of the E2F-1 recognition sequence in the *hTERT* promoter, thus leading to increased binding of E2F-1 to its promoter and therefore repressing *hTERT* and telomerase activity.

Resveratrol (Figure 3F), which is contained in grapes, red wine, peanuts, and some berries, possesses preventive effects against cancer, cardiovascular diseases, and neurodegenerative disorders [75]. Lanzilli et al. [76] found that resveratrol reduces telomerase activity and the nuclear protein levels of hTERT in MCF-7 cells. Pterostilbene, a dimethyl ether analog of resveratrol, has telomerase-inhibitory properties [77]. Kala et al. [78] showed that a combination of resveratrol (15 μM) and pterostilbene (5 μM) decreases *hTERT* expression through sirtuin 1 (SIRT1) and DNMT inhibition in HCC1806 breast cancer cells. The knockdown of SIRT1, a nicotinamide adenine dinucleotide-dependent deacetylase, represses *hTERT* mRNA expression, suggesting the involvement of SIRT1 in *hTERT* regulation.

### 2.4. Ceramide

This compound (Figure 3G) consists of sphingosine, a long-chain amino alcohol, linked to a fatty acid via an amide bond, and plays a major part in the metabolism of sphingolipids. Ceramide is used as a substrate to generate more complex sphingolipids (such as sphingomyelin and glycosphingolipids, which are essential components of the cell membrane) while being a degradation product of complex sphingolipid molecules [79]. Ceramide can regulate various cellular phenomena, including cell proliferation, death, migration, and senescence [80]. Inhibitory effects of ceramide on telomerase in A549 human lung adenocarcinoma cells have been extensively investigated by Ogretmen et al. [81,82,83,84,85]. For example, treatment with 20 μM C6-ceramide suppresses cellular telomerase activity and causes shortening of telomere length, whereas 20 μM dihydro-C6-ceramide shows no inhibition [81,82]. Overexpression of sphingomyelinase, the enzyme responsible for the conversion of sphingomyelin to ceramide, significantly decreases telomerase activity. Moreover, overexpression of glucosylceramide synthase, which converts ceramide to glucosylceramide, attenuates the inhibitory effects of C6-ceramide on telomerase. These findings suggest that telomerase inhibition is specific to ceramide, but not to other sphingolipids. Ceramide represses telomerase activity via decreased *hTERT* mRNA expression by rapid degradation of the c-Myc protein [83]. Wooten and Ogretmen [84] revealed that downregulation of *hTERT* mRNA by ceramide is mediated by Sp1 and Sp3 (transcription factors of the Sp1 family)-dependent regulation of *hTERT* transcription. Ceramide treatment elicits deacetylation of Sp3 and histone H3 at an *hTERT* promoter site, thereby preventing the transcription of *hTERT* [85].

### 2.5. Sulfoquinovosyldiacylglycerol (SQDG)

The photosynthetic membranes of higher plants contain abundant glyceroglycolipids, including SQDG (Figure 3H), monogalactosyldiacylglycerol (MGDG), and digalactosyldiacylglycerol (DGDG) [86]. SQDG possesses a unique sulfoquinovose headgroup, a derivative of glucose with a sulfonate group, and exerts anticancer effects [87]. We demonstrated that SQDG directly inhibits telomerase activity with 50% inhibition at 22 μM, whereas MGDG and DGDG have no effect even at concentrations of 100 μM [88]. Eicosapentaenoic acid (EPA), one of SQDG components, inhibits this enzymatic activity too, suggesting that the structure of the sulfate group and fatty acid of SQDG is essential for the inhibitory action on telomerase activity.

### 2.6. Fatty Acids

Fatty acids are a fundamental component of dietary lipids. Among polyunsaturated fatty acids, the n-3 and n-6 fatty acids are distinguished by the positions of double bonds with respect to the methyl end of the acyl chain. For example, in n-3 fatty acids, the first double bond is located at the third carbon atom from the methyl end. Both n-3 and n-6 fatty acids are important constituents of cell membrane lipids and therefore influence cell membrane properties such as fluidity, flexibility, and membrane-bound enzymatic activities [89]. In addition to the structural functions, n-3 and n-6 fatty acids can act as precursors of bioactive lipid mediators [90]. Long-chain n-3 fatty acids such as EPA (Figure 3I) and docosahexaenoic acid (DHA) have protective effects against inflammation, cancer, cardiovascular disease, and dementia [91]. We found that fatty acids (C18–C22) directly inhibit telomerase activity [92]. IC_50_ (the concentration causing 50% inhibition of the telomerase activity) values are listed in Table 2, indicating that the inhibitory potency of fatty acids increases with the number of double bonds, and that *cis*-fatty acids possess higher inhibitory activities than *trans*-isomers do. Accordingly, polyunsaturated fatty acids such as EPA and DHA can strongly prevent telomerase enzymatic activity. A Lineweaver–Burk plot revealed that EPA is a competitive inhibitor relative to the telomerase substrate primer, implying that fatty acids may interact with the primer-binding site of telomerase. Besides, we demonstrated that physiological concentrations of EPA and DHA (≤50 μM) downregulate *hTERT* and *c-myc* mRNA via PKC inhibition, thereby repressing telomerase activity. Our results indicate that fatty acids, especially EPA and DHA, not only directly inhibit the enzymatic activity of telomerase but also downregulate telomerase at the transcriptional level.

### 2.7. Tocotrienol

Vitamin E forms can be categorized into two groups: tocopherol (Toc) and tocotrienol (T3), based on their structural difference in the isoprenoid-derived hydrophobic tail (Figure 3J). Both Toc and T3 have four forms: α-, β-, γ-, and δ-isomers. Toc is contained in a variety of foods (e.g., nuts, whole grains, and common vegetable oils). In contrast, T3 is present at low levels in most plants but annatto, palm, and rice bran oils are richer sources of T3 [93]. T3 has a broad range of beneficial activities, including antioxidative, antitumor, antidiabetic, anti-inflammatory, cardioprotective, and neuroprotective properties [94]. These bioactivities of T3 are superior to those of Toc because incorporation of T3 into the lipid bilayer of the cell membrane is more effective than that of Toc [95]. We revealed that 5–20 μM T3, particularly β- and δ-isomers, dose-dependently suppresses telomerase activity in DLD-1 human colorectal adenocarcinoma cells, and the inhibitory potency of δ-T3 is stronger than that of the β-isomer [96]. On the other hand, Toc (α-, β-, γ-, and δ-isomers) exerts only negligible inhibition of telomerase. The reduction in cellular telomerase activity by T3 is due to downregulation of *hTERT* and *c-myc* mRNA through PKC inhibition. Furthermore, ferulic acid, a potent phenolic antioxidant abundant in rice bran oil, potentiates the anticancer effects of T3 [97]. Co-treatment with T3 and ferulic acid therefore synergistically decrease telomerase and *hTERT* expression [98,99].

### 2.8. Sulforaphane

This compound (Figure 3K) is a sulfur-containing isothiocyanate derivative present in cruciferous vegetables such as broccoli, broccoli sprouts, and cauliflower. Sulforaphane exerts powerful anticancer activity via apoptosis induction, suppression of cell cycle progression, angiogenesis inhibition, and anti-inflammatory action [100]. Moon et al. [101] found that 10–20 μM sulforaphane suppresses telomerase activity in Hep3B human hepatoma cells. The reduction in telomerase activity is mediated by downregulation of *hTERT* mRNA via c-Myc suppression, as well as dephosphorylation of hTERT through Akt inhibition. Meeran et al. [102] reported that treatment of breast cancer cells (MCF-7 and MDA-MB-231) with sulforaphane causes demethylation of the *hTERT* promoter via DNMT inhibition, thereby facilitating the binding of CTCF to the promoter site and attenuating *hTERT* expression and telomerase activity.

## 3. Telomerase Induction in Cancer Cells by Dietary Factors

### 3.1. Genistein

As described above, genistein in the pharmacological concentration range (10–100 μM) reduces telomerase activity. Because physiological concentrations of genistein are estimated to be <2 μM, Chau et al. [103] investigated the effect of genistein on telomerase at concentrations of ≤1 μM. As a result, physiologically achievable concentrations of genistein augmented telomerase activity and *hTERT* mRNA expression in DU-145 cells. Of note, TRAMP mice—a popular transgenic mouse model of prostate cancer—fed a diet containing genistein (250 mg/kg diet) from 12 to 20 weeks of age demonstrated a threefold increase in prostate weight as compared with the control group. Moreover, significant elevation of telomerase activity was observed in the prostate tissue of TRAMP mice consuming the genistein-supplemented diet. This study raised the possibility that physiological concentrations of genistein may have adverse effects on patients with prostate cancer.

### 3.2. A Glycated Lipid

Maillard reactions may contribute to food deterioration and the pathogenesis of diabetes. Our research group found that phosphatidylethanolamine (PE) reacts with glucose to form a PE-linked Amadori product (Amadori-PE; Figure 3L) [104]. Amadori-PE is present in some foods (e.g., infant formula and chocolate) [105] and, moreover, crucially participates in the development of diabetic complications [106]. Several epidemiological studies have shown that type 2 diabetes significantly raises cancer risk [107]. Nevertheless, little is known about the molecular mechanism underlying the involvement of diabetes in tumor development. We, therefore, examined the link between Amadori-PE and tumor progression, with an emphasis on telomerase activity. Physiological concentrations of Amadori-PE (1–5 μM: plasma concentrations of this lipid in diabetic patients) increased telomerase activity in PANC-1 human pancreatic carcinoma cells by upregulating *hTERT* mRNA expression through the induction of *c-myc* [108]. A similar phenomenon was observed in other cancer cells (MIA PaCa-2 human pancreatic carcinoma, HepG2 human hepatoma, and DLD-1). This finding provides experimental evidence for a unique role of lipid glycation in the relation between diabetes and cancer.

## 4. Conclusions

Establishment of the method for the sensitive detection of telomerase activity, namely, the telomeric repeat amplification protocol (TRAP) assay [4], in 1994 enabled screening for telomerase inhibitors, as well as the diagnosis of cancer by means of telomerase. Since then, numerous types of telomerase inhibitors have been identified and developed. Examples of such inhibitors include reverse transcriptase inhibitors (e.g., 3′-azido-3′-deoxythymidine triphosphate [109]), hTR antisense oligonucleotides (e.g., imetelstat [110]), G-quadruplex stabilizers (for instance, TMPyP4 [111]), synthetic compounds from random screening (e.g., BIBR1532 [112]), and HSP90 inhibitors (such as geldanamycin [113]). Among these inhibitors, imetelstat is a potent agent specific to telomerase. Imetelstat can bind with high affinity to hTR, thus blocking the interaction between telomerase and telomere DNA. Recent phase II trials uncovered a clinical benefit of imetelstat for patients with myeloproliferative disorders [114,115].

As mentioned above, several dietary components (i.e., RA, 1α,25(OH)_2_VD_3_, polyphenols, ceramide, SQDG, fatty acids, tocotrienol, and sulforaphane) inhibit telomerase by directly blocking the enzymatic activity and/or by decreasing *hTERT* expression. Most telomerase inhibitors derived from food factors have been evaluated only in cell culture experiments. To date, a few studies have focused on the in vivo efficacy of these inhibitors [54,59]. Orally administered EGCG suppresses tumor size and shortens telomere length in tumor tissue [59], indicating that EGCG exhibits the telomerase-inhibitory effect in vivo. The in vivo anticancer efficacy of 1α,25(OH)_2_VD_3_ combined with 9-*cis*-retinoic acid has been examined only by tumor volume measurement [54]. It is unclear whether the tumor suppressive effect of 1α,25(OH)_2_VD_3_ and 9-*cis*-retinoic acid is mediated through telomerase inhibition since VDR-RXR can affect diverse growth-regulatory signaling pathways [116]. The evaluation of telomerase activity, telomere length, *hTERT* expression, or their combination, as well as tumor size measurement, would be necessary to determine the in vivo effectiveness of telomerase inhibitors. In contrast to the chemically synthesized inhibitors such as imetelstat, a human trial targeting telomerase using dietary compounds has never been conducted. A clinical application of dietary factors to cancer treatment is highly attractive because they are nontoxic to healthy cells at physiological concentrations. Prior to initiating human clinical trials, further animal experiments should be performed to investigate their potential applications in cancer treatment and prevention. On the other hand, genistein and Amadori-PE induce telomerase activity in cancer cells. Telomerase activity levels in tumor cells positively correlate with an aggressive tumor phenotype [117]. Overexpression of *hTERT* induces cell proliferation via upregulation of growth-controlling genes and promotes cell survival by apoptosis suppression [118,119]. Therefore, telomerase activation by genistein and Amadori-PE may be relevant to cancer progression.

The TRAP assay is highly sensitive but tends to generate several extra bands, raising the possibility that the potency of telomerase inhibitors may be inappropriately evaluated [120]. However, this assay has been applied by many research groups [46,55,58,66,73,76,81,101,103]. Thus, some modified TRAP assays have been reported with improved specificity and reliability [121]. Tatematsu et al. [122] developed a stretch PCR method that achieves quantitative evaluation by the addition of extra tag sequences to the 5′-ends of the forward and reverse PCR primers, and we and some researchers have utilized this method [54,88,92,96,98,108]. On the other hand, treatment with EGCG [58], curcumin [69], and ceramide [82] leads to telomere shortening in cancer cells. Multiple methods have been developed for telomere length analysis, including terminal restriction fragment (TRF) analysis, real-time PCR, single telomere length analysis, and quantitative fluorescence in situ hybridization [123]. Among these methods, TRF analysis is widely used [58,69,82] and is often regarded as the gold standard.

A more detailed understanding of the mechanisms underlying telomerase regulation will be necessary for the identification of novel telomerase inhibitors. Further studies, with special emphasis on in vivo experiments, are needed to apply dietary compounds to cancer treatment and prevention.

## Figures and Tables

**Table 1:** *hTERT* regulation by miRNA.

Tumor Type	miRNA	Reference
Neuroblastoma	miR-138	[33]
Anaplastic thyroid carcinoma	miR-138	[34]
Gastric cancer	miR-1207-5p	[35]
	miR-1266	[35]
	miR-1182	[36]
Cervical cancer	miR-491-5p	[37]
Head and neck squamous cell carcinoma	miR-512-5p	[38]
Ovarian cancer	miR-532	[39]
	miR-3064	[39]
Breast cancer	miR-296	[40]
	miR-512	[40]

**Table 2:** IC_50_ values of various fatty acids.

Number of Carbon Atoms	Fatty Acid	IC_50_ (μM) ^1^
C12	Lauric acid [12:0]	>100
	*cis*-11-Dodecenoic acid [12:1 Δ11*cis*]	>100
C14	Myristic acid [14:0]	>100
	Myristoleic acid [14:1 Δ9*cis*]	>100
C16	Palmitic acid [16:0]	>100
	Palmitoleic acid [16:1 Δ9*cis*]	25
C18	Stearic acid [18:0]	>100
	Oleic acid [18:1 Δ9*cis*]	35
	Vaccenic acid [18:1 Δ11*cis*]	38
	Elaidic acid [18:1 Δ9*trans*]	55
	*trans*-Vaccenic acid [18:1 Δ11*trans*]	74
	Linoleic acid [18:2 Δ9-12*cis*]	25
	Linolelaidic acid [18:2 Δ9-12*trans*]	>50
	γ-Linolenic acid [18:3 Δ6-9-12*cis*]	13
	α-Linolenic acid [18:2 Δ9-12-15*cis*]	10
C20	Arachidic acid [20:0]	>100
	*cis*-5-Eicosenoic acid [20:1 Δ5*cis*]	>100
	*cis*-11-Eicosenoic acid [20:1 Δ11*cis*]	>100
	*trans*-11-Eicosenoic acid [20:1 Δ11*trans*]	>100
	*cis*-11-14-Eicosadienoic acid [20:2 Δ11-14*cis*]	70
	*cis*-8-11-14-Eicosatrienoic acid [20:3 Δ8-11-14*cis*]	30
	*cis*-11-14-17-Eicosatrienoic acid [20:3 Δ11-14-17*cis*]	24
	Arachidonic acid [20:4 Δ5-8-11-14*cis*]	25
	Eicosapentaenoic acid (EPA) [20:5 Δ5-8-11-14-17*cis*]	19
C22	*cis*-13-16-19-Docosatrienoic acid [22:3 Δ13-16-19*cis*]	45
	Docosahexaenoic acid (DHA) [22:6 Δ4-7-10-13-16-19*cis*]	5

^1^ IC_50_: concentration (μM) causing 50% inhibition of telomerase activity.

## Abbreviations

hTR	human telomerase RNA component
hTERT	human telomerase reverse transcriptase
AP-2	activating enhancer-binding protein-2
HIF-1	hypoxia-inducible factor 1
ER	estrogen receptor
AP-1	activator protein 1
VDR	vitamin D receptor
RXR	retinoid X receptor
WT1	Wilms’ tumor 1
MZF-2	myeloid zinc finger protein 2
CTCF	CCCTC-binding factor
DNMT	DNA methyltransferase
HAT	histone acetyltransferase
HDAC	histone deacetylase
TSA	trichostatin A
SMYD3	SET and MYND domain-containing protein 3
H3K4	lysine 4 on histone 3
miRNA	microRNA
3′UTR	3′ untranslated region
RA	all-*trans* retinoic acid
RAR	retinoic acid receptor
CYP	cytochrome P450
1α,25(OH)_2_VD_3_	1α,25-dihydroxyvitamin D_3_
VDRE	vitamin D response element
EGCG	(−)-epigallocatechin-3-gallate
Hsp90	heat shock protein 90
PKC	protein kinase C
SIRT1	sirtuin 1
SQDG	Sulfoquinovosyldiacylglycerol
MGDG	monogalactosyldiacylglycerol
DGDG	digalactosyldiacylglycerol
EPA	eicosapentaenoic acid
DHA	docosahexaenoic acid
Toc	tocopherol
T3	tocotrienol
PE	phosphatidylethanolamine
Amadori-PE	phosphatidylethanolamine-linked Amadori product
TRAP	telomeric repeat amplification protocol
